# The Restoring Effect of Human Umbilical Cord-Derived Mesenchymal Cell-Conditioned Medium (hMSC-CM) against Carbon Tetrachloride-Induced Pulmonary Fibrosis in Male Wistar Rats

**DOI:** 10.1155/2022/7179766

**Published:** 2022-12-22

**Authors:** Maryam Khajvand-Abedini, Mahdi Bahmani, Nasrin Ziamajidi, Alireza Nourian, Parisa Habibi, Shirin Heidarisasan, Roghayeh Abbasalipourkabir

**Affiliations:** ^1^Department of Biochemistry, School of Medicine, Hamadan University of Medical Sciences, Hamadan, Iran; ^2^Department of Pathology, School of Veterinary Medicine Clinical Biochemistry, University of Bu-Ali Sina, Hamadan, Iran; ^3^Department of Physiology, School of Medicine, Hamadan University of Medical Sciences, Hamadan, Iran

## Abstract

**Objective:**

Pulmonary toxicity induced by CCl_4_, a model of idiopathic pulmonary fibrosis (IPF), leads to tissue remodeling and inflammation. Human umbilical cord mesenchymal cell-conditioned medium (hMSC-CM) is a potent anti-inflammatory, antioxidative, and antifibrotic agent.

**Methods:**

Forty male Wistar rats were assigned to the control (C), olive oil control (C.O) (hMSC-CM), control (C.Ms), fibrosis (fb), and fibrosis with hMSC-CM (f.Ms) treatment groups. The groups C, C.O, and C.Ms received PBS (200 *µ*l), olive oil (1 ml/kg), and hMSC-CM (100 *μ*g protein/kg), respectively. The fibrosis group was administered with only CCl4 (1 ml/kg). The last group, f.Ms was treated with CCl_4_ (1 ml/kg) and 100 *μ*g protein/kg IV hMSC-CM. While the treatment with olive oil and CCl_4_ was performed for 2 days/week from the first week for 12 weeks, the treatment with PBS and hMSC-CM was carried out 2 days/week from week 4^th^ to week 12^th^. The effect of the UC-MSC culture medium treatment on the lung was evaluated by assessing lysyl oxidase (LOX), tumor necrosis factor-alpha (TNF-*α*), and transforming growth factor-*β*1 (TGF-*β*1) genes, and proteins expression by real-time RCR and western blotting, respectively.

**Results:**

Lysyl oxidase (LOX), tumor necrosis factor-alpha (TNF-*α*), transforming growth factor-b1 (TGF-*β*1), malondialdehyde (MDA), and oxidative stress levels were markedly higher in the fibrosis group than in the control groups (*p* ≤ 0.001). Additionally, glutathione (GSH) in the fibrosis group was markedly lower than those in the control groups (*p* ≤ 0.001). Fibrosis in the UC-MSC treatment group had milder histopathological injuries than in the fibrosis group.

**Conclusion:**

hMSC-MSC as a strong anti-inflammatory, antioxidative, and antifibrotic decreases the level of oxidative stress, proinflammatory cytokines, and MDA causing a restoring effect against CCl_4_-induced pulmonary fibrosis.

## 1. Introduction

Idiopathic pulmonary fibrosis (IPF), such as chronic lung disease, is characterized by the excessive accumulation of myofibroblasts and extracellular matrix (ECM) proteins in the lung, which leads to compromised lung function and increased lung remodeling[1]. Despite the repair of damaged alveolar epithelial cells by inflammatory and growth factors, dysregulation of the repair process leads to lung fibrosis [2]. Based on the evidence, proinflammatory factors, growth factors, and collagen deposition generated from fibroblasts causes IPF. Accordingly, the cross-linking reaction and deposition of collagen are catalyzed by lysyl oxidase (LOX) [3]. On the other hand, the release of inflammatory cytokines such as tumor necrosis factor-alpha (TNF-*α*) and other ligands such as transforming growth factor-*β*1 (TGF-*β*1) have regular effects on LOX levels [4]. There is ample research on the role of TGF-*β*1 in the increase in LOX levels by multiple signaling pathways and factors [3, 5].

In addition, pulmonary fibrosis can be induced by the exposure to chemotherapy, radiotherapy, drugs, and environmental toxins such as carbon tetrachloride (CCl_4_) which have toxic effects. The harmful effects of CCl_4_ are caused by the trichloromethyl (CCl_3_^●^) and trichloromethyl peroxyl (CCl_3_O_2_^●^) radicals. Therefore, CCl_4_ radicals are known to cause lung fibrosis by lowering glutathione (GSH) and elevating lipid peroxidation, releasing cytokines such as TNF-*α* and growth factors such as TGF-*β*1. Thus, the released mediators cause increased mRNA LOX expression, fibroblastic proliferation, and ECM deposition [5–7].

Despite the lack of specific treatment methods for IPF, lung transplantation and corticosteroid administration are common treatments for IPF. Although the side effects of corticosteroid therapy, such as hyperglycemia, hypertension, and electrolyte anomalies are well known [8], novel therapeutic methods with minimal side effects are needed [9, 10]. Recent reports have shown the administrations of MSC (mesenchymal stem cells) as effective agents on lung repair for animal models with IPF [11–13]. Accordingly, in the ideal time of MSC therapy after the establishment of the IPF models (6–90 days), TNF-*α* and TGF-*β*1 levels returned to normal levels [8, 14–16]. According to the literature [17], MSC specializations could be better identified at most available MSC resources for therapeutic strategies; thus, we used human umbilical cord mesenchymal cell-conditioned medium (hMSC-CM) for the experiments. Current studies have mostly used the IPF model induced by bleomycin [18, 19], and a few studies have used the IPF model induced by CCl_4_ [7, 20, 21]. To determine whether MSCs have a medicinal effect on IPF in rats, this study established an IPF model using CCl_4_ in rats and assessed the effects of intravenous UC-MSC culture medium transplantation.

## 2. Methods

### 2.1. The Preparation and Intravenous Transplantation of hMSC-CM

The culture, isolation, and purity evaluation of UC-MSC from human infant umbilical cord samples were conducted according to preliminary studies [22, 23]. Briefly, after the human umbilical cord mesenchymal cells were cultured in DMEM-low glucose for 72 h, the detached and suspended cells in the fresh medium were centrifuged at 400*g* and 15000*g* for 10 min, respectively. The supernatant obtained was used to treat the animals with pulmonary fibrosis. This study was approved by the Research Ethics Committee of Hamadan University of Medical Sciences (IR.UMSHA.REC.1398.1027). To assess the side effects of three doses of 100, 200, and 400 *µ*g protein/kg/body weight by biochemical and histopathological observations, the 100 *µ*g protein/kg/body weight dosage of hMSC-CM was equivalent to the impressive dose used in relevant investigations [17].

### 2.2. Animal Study

Forty male Wistar rats weighing 180–200 g were acquired from the animal house of Hamadan University of Medical Sciences and maintained under standard conditions (22–24°C temperature, 12/12-hour light/dark cycle, free access to food and water). Considering the 12-week period for this study, the animals were randomly divided into five groups (*n* = 8) as follows:

1. Group C (control group): the animals received sterile PBS (100 *μ*l/IV, 2 days/week, beginning from the 4^th^ week during the 12 weeks of the experiment).
2. Group C.O (olive oil control group): the animals received intraperitoneal olive oil (1 ml/kg/IP, 2 days/week, from the first week during 12 weeks of the experiment) and received sterile PBS intravenously, as in group C.
3. Group C.Ms (hMSC-CM control group): the animals received hMSC-CM culture medium (100 *μ*g protein/kg, intravenously through the tail vein with an insulin syringe, 2 days/week, from the 4^th^ week during the 12 weeks of the experiment).
4. Group Fb (fibrosis group): the animals received intraperitoneal CCl_4_ at the first and third days of each week (1 ml/kg, diluted in olive oil (1 : 1), IP, 2 days/week, starting the first week during 12 weeks of the experiment.
5. Group Fb.Ms (group Fb with the hMSC-CM treatment): the animals received CCl_4_ like group Fb and hMSC-CM culture medium like group C.Ms (24 h after each treatment with CCl_4_).

### 2.3. Sampling

Twenty-four hours after the last treatment, all rats were sacrificed. Subsequently, lung tissues were separated and divided into two parts. The first part of the lungs was taken for histopathologic examination, while the second part was stored at −80°C for other investigations.

### 2.4. Histopathological Investigation

Lung samples were fixed in 10% buffered formalin, embedded in paraffin, and sliced into 5 *μ*m thick sections. The sections were then stained with hematoxylin and eosin (H & E) and Masson's trichrome (M'sT). Finally, the stained sections were studied using a light microscopic view of the lung tissue from different groups of rats for the demonstration of collagen.

### 2.5. The Preparation of Lung Lysates for Biochemical Assays

Lung tissues were washed in ice-cold PBS or phosphate-buffered saline (pH 7.4) and were powdered using liquid N_2_. Then, the powdered tissue was homogenized with lysis buffer (1.5 mM MgCl_2_, 1 mM EDTA, 10 mM HEPES, 10 mM KCl, and 0.1% Triton X100 (at pH 7.9)) and protease inhibitor cocktail (Sigma-Aldrich, US). After shaking (30 min, 4°C) and centrifugation (12,000 × *g*, 20 min, 4°C), the cell-free supernatant was separated immediately. The total protein level of the supernatants was assayed by the Bradford method using bovine serum albumin (BSA) as a standard protein [24].

### 2.6. Total Antioxidant Capacity (TAC) and Total Oxidant Status (TOS) Assays

The total antioxidant capacity (TAC) assay was conducted using the ferric reducing antioxidant power method (FRAP) [25], while the total oxidative capacity was determined by the oxidation of ferrous iron to ferric in lung tissues and the measurement of ferric by xylose orange [26]. The division of TOS into TAC was performed to compute the oxidative stress index (OSI).

### 2.7. Glutathione (GSH) and Malondialdehyde (MDA) Assays

MDA and GSH in the lung tissues were measured according to the instructions of the assay kit producer (Kiazist, Iran).

### 2.8. Hydroxyproline Contents Assay

The method described in a previous study [22] was applied to the hydroxyproline content assay of the lung samples from each rat. Lung tissues were powdered using liquid N_2_ and 30 mg of each tissue was transferred into a micro tube. Then, 100 *µ*l dH_2_O was added to each tube and micro tubes were vortexed. After that, 100 *µ*L HCl 12 M was added to the tubes and the tubes were vortexed. Next, the tubes were incubated for 48 h at 120°C. The dried samples were recovered in 200 *μ*l assay buffer and centrifuged at 12,000 × *g* for 15 min. Finally, 50 ml of oxidant was added to the supernatant and incubated for 15 min at room temperature. After reacting supernatants with Ehrlich's reagent, according to the instructions of the assay kit producer (Kiazist, Iran), colored products as hydroxyproline content indexes were measured via spectrophotometry at 540 nm.

### 2.9. Enzyme-Linked Immunosorbent Assay (ELISA)

After the lung homogenate was prepared, the protein levels of TNF-*α* (pg/mg protein) were assayed using a rat TNF-*α* ELISA MAX™ Deluxe set kit (BioLegend, San Diego, USA) and subsequently normalized to the total protein content.

### 2.10. Western Blot Assay

The homogenate of the lung tissue was placed in radio-immunoprecipitation assay (RIPA) lysis buffer containing a mixed protease inhibitor to obtain total proteins. Protein concentrations in the homogenates were assayed using the bicinchoninic acid (BCA) method. Specific amounts of the protein were separated by 10% SDS-PAGE, transferred to PVDF membranes (Millipore, Billerica, United States), and blocked with 5% BSA for 2 h before incubation with primary antibodies against TGF-*β*1 (ab215715, Abcam), LOX (ab174316, Abcam), and *β*-actin (ab119716, Abcam) as loading controls for 14 h at 4°C. After washing with Tris-buffered saline (TBST), the membranes were incubated with HRP-labeled goat anti-rabbit IgG for 1 h at room temperature, and the bound antibody was detected using an enhanced chemiluminescence detection reagent (Bio-Rad, Hercules, CA, United States). The protein expression was quantified by the densitometric analysis by ImageJ software [27].

### 2.11. Molecular Investigation of Lung Tissue

The purity and concentration of extracted total RNA (using TRIzol reagent, Invitrogen™, Thermo Fisher Scientific, United States) were confirmed using a Nanodrop 2,000 spectrophotometer (Thermo Fisher Scientific, MA, United States). cDNA synthesis (Gene All kit, Seoul, South Korea) and real-time PCR were conducted using SYBR Green master mix (Amplicon, Odense, Denmark) and Roche LightCycler® 96 system (Roche, Germany), according to the manufacturer's protocols. All data were analyzed using the 2^−ΔΔCt^ method. The primer sequences used are listed in Table 1.

### 2.12. Statistical Analysis

Statistical analyses were conducted using SPSS software (version 16.0; IBM, Armonk, NY, USA) and GraphPad Prism 8.00 software (LaJolla, CA, USA). For multiple comparisons among notice groups, statistical assays were performed by one-way analysis of variance (ANOVA) and post-hoc Tukey test with *p*-value <0.05 indicating the significance level.

## 3. Results

### 3.1. Histopathological Findings

**Figure 1:**
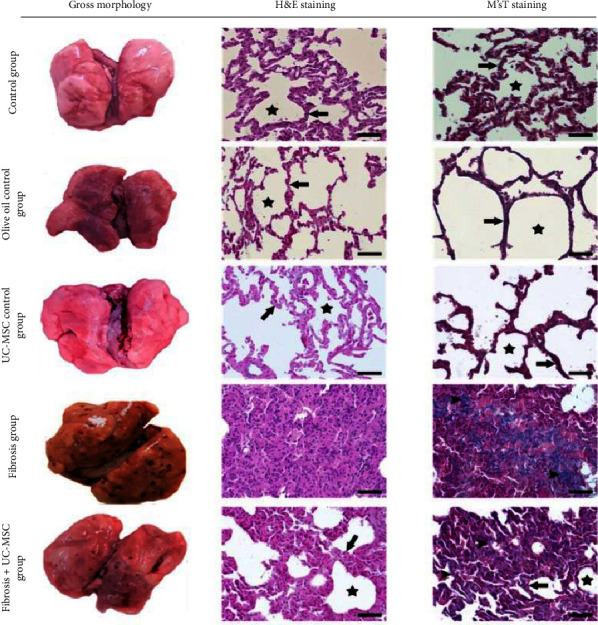
The results of histological examination of the lung tissue with H&E and M'sT staining; (magnification = 400x, scale bar = 50 *μ*m). C: control group, C.O: olive oil control group. C.Ms: hMSC-CM control group, Fb: fibrosis group, Fb.Ms: group Fb with hMSC-CM treatment. The black arrow (**↑**) shows the alveolar wall, the black asterisk (*∗*) shows alveoli, and the black arrowhead (**►**) shows collagen deposition.

As shown in Figure 1, rats that received normal saline in group C had a normal architecture of the lung tissue, typical alveoli, and thin alveolar walls. Furthermore, the administration of olive oil and MSCs in groups C.O and C.Ms did not affect the normal histology of the organ (the alveoli had thin walls with no evidence of fibrosis).

In contrast, the lungs of rats administered with CCl_4_ in group Fb showed extensive fibrosis with widespread deposition of collagen fibers throughout the organ. The interstitial tissue proliferation led to the alteration of organ architecture, so few alveoli, if any, could be detected in the tissue. Nevertheless, rats that received CCl_4_ together with MSC in the Fb.Ms group showed reduced fibrotic tissue mass, expansion of alveolar spaces, and narrowing of alveolar walls.

### 3.2. Biochemical Investigation

**Figure 2:**
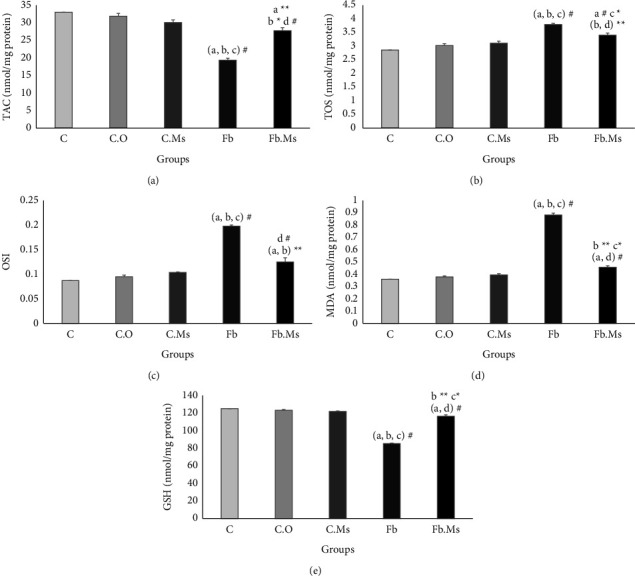
The results of the oxidative stress markers in the lung tissue of the studied animals. (a) Total antioxidant capacity (TAC), (b) total oxidant status (TOS), (c) oxidative stress index (OSI), (d) malondialdehyde (MDA), (e) glutathione (GSH), C: control group; C.O: olive oil control group. C.Ms: hMSC-CM control group, Fb: fibrosis group, Fb.Ms: group Fb with hMSC-CM treatment. (^*∗*^*p* < 0.05, ^*∗∗*^*p* < 0.01, ^#^*p* < 0.001). ^a^Significantly different from C. ^b^Significantly different from C.O. ^c^Significantly different from C.Ms. ^d^Significantly different from that of Fb.

In group Fb, the mean levels of TOS, OSI, and MDA increased (Figures 2(a)–2(c), *p* < 0.001) whereas TAC and GSH levels decreased significantly (Figures 2(d) and 2(e), *p* < 0.001) in comparison with groups C, C.O, and C.Ms. However, the TOS (Figure 2(a), *p* < 0.01), OSI, and MDA (Figures 2(b) and 2(c), *p* < 0.001) levels were lower in the Fb.Ms group than in the Fb group. Also, TAC and GSH levels increased significantly in the Fb.Ms group compared to those in the Fb group (Figures 2(d) and 2(e), *p* < 0.001).

After comparing the group Fb.Ms with group C, a significant increase was observed in the TOS (Figure 2(a), *p* < 0.001), OSI (Figure 2(b), *p* < 0.01), and MDA (Figure 2(c), *p* < 0.001) levels. Additionally, the group Fb.Ms exhibited a notable increase in TAC (Figure 2(d), *p* < 0.01) and GSH (Figure 2(e), *p* < 0.001) compared to group C. Moreover, the TOS, OSI, and MDA (Figures 2(a)–2(c), *p* < 0.01) levels significantly increased, and TAC (Figure 2(d), *p* < 0.05) and GSH (Figure 2(e), *p* < 0.01) were significantly lower in group Fb.Ms than in group C.O. Furthermore, the TOS and MDA levels significantly increased, and the GSH level significantly decreased in group Fb.Ms compared to the group C.Ms (Figures 2(a), 2(c) and 2(e), *p* < 0.05).

### 3.3. Hydroxyproline Content

**Figure 3:**
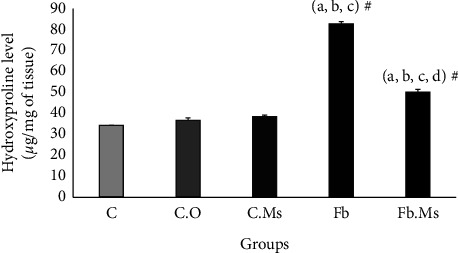
Hydroxyproline content in the lung tissue of the studied animals. C: control group; C.O: olive oil control group. C.Ms: hMSC-CM control group, Fb: fibrosis group, Fb.Ms: group Fb with hMSC-CM treatment. (^#^*p* < 0.001). ^a^Significantly different from C. ^b^Significantly different from C.O. ^c^Significantly different from C.Ms. ^d^Significantly different from that of Fb.

As shown in Figure 3, pulmonary hydroxyproline content was remarkably increased in rats of group Fb compared with groups C, C.O, and C.Ms (*p* < 0.001). Furthermore, the pulmonary hydroxyproline content was significantly lower in Fb.Ms than in the group Fb (*p* < 0.001) but was augmented compared with groups C, C.O, and C.Ms (*p* < 0.001).

### 3.4. The Gene Expression and Protein Levels of LOX, TGF-*β*1, and TNF-*α*

**Figure 4:**
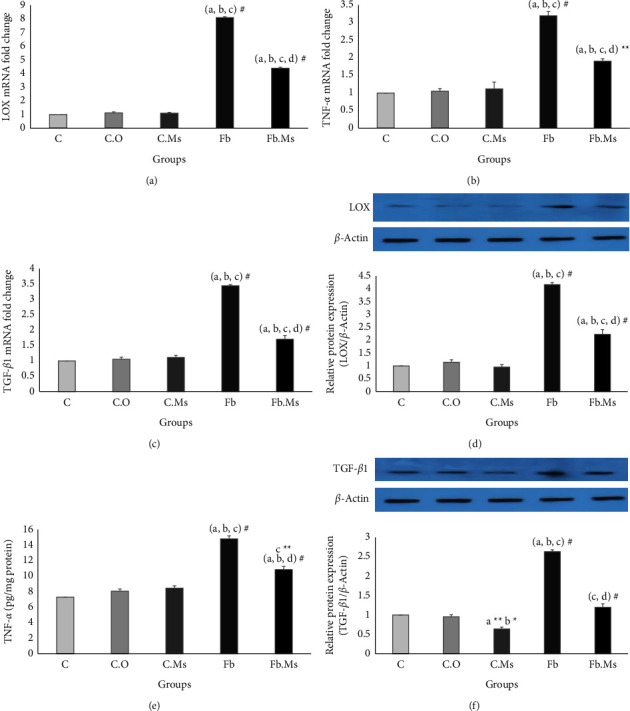
The results of the protein and gene expressions of LOX, TNF-*α*, and TGF-*β*1 in the lung tissue of studied animals. (a) Level expressions of lysyl oxidase (LOX) gene, (b) level expressions of tumor necrosis factor-alpha (TNF-*α*) gene, (c) level expressions of transforming growth factor-*β*1 (TGF-*β*1) gene. (d) Level expressions of LOX protein. (e) Level expressions of TNF-*α* protein, (f) level expressions of TGF-*β*1 protein. C: control group; C.O: olive oil control group. C.Ms: hMSC-CM control group, Fb: fibrosis group, Fb.Ms: group Fb with hMSC-CM treatment. (^*∗*^*p* < 0.05, ^*∗∗*^*p* < 0.01, ^#^*p* < 0.001). ^a^Significantly different from C. ^b^Significantly different from C.O. ^c^Significantly different from C.Ms. ^d^Significantly different from that of Fb.

The gene expression levels of LOX, TGF-*β*1, and TNF-*α* in different groups by RT-PCR agreed with the western blot results. As shown in Figure 4, compared with groups C, C.O, and C.Ms, the gene expression and protein levels of LOX, TNF-*α,* and TGF-*β*1 were notably higher in group Fb (Figures 4 and 4(e), *p* < 0.001). Although, the treatment with hMSC-CM significantly prevents increasing gene expression levels of LOX, TNF-*α*, and TGF-*β*1 in group Fb.Ms in comparing with group Fb, the levels of these variables in the group Fb.Ms still was remarkably higher than in groups C, C.O, and C.Ms (*p* < 0.001, *p* < 0.01, and *p* < 0.001, respectively, Figures 4(a)–4(c)). The treatment with hMSC-CM in the group Fb.Ms also significantly decreased the protein level of LOX, TNF-*α,* and TGF-*β*1 versus group Fb (*p* < 0.001), where the protein level of LOX (in comparing with the control groups, *p* < 0.001), TNF-*α* (in comparing with the groups C and C.O, *p* < 0.001; and C.Ms, *p* < 0.001), and TGF-*β*1 (versus group C.Ms, *p* < 0.001) were still significantly high (Figures 4(d)–4(f)).

## 4. Discussion

In our study, the OSI and MDA levels, gene expression, and protein levels of LOX, TGF-*β*1, and TNF-*α* in the lung tissue of the CCl_4_ administered group were clearly higher than those in the control groups. Histopathological investigations of the tissues in group Fb showed serious histopathological injuries, such as the widespread deposition of collagen fibers, lack of alveoli, and excessive lung tissue injury. In contrast, OSI and MDA levels, gene expression, and protein levels of LOX, TGF-*β*1, and TNF-*α* in the Fb.Ms group were noticeably lower than those in the Fb group. In the above-mentioned group, histopathological damage in the lung tissues improved, the fibrotic tissue mass shrank, and alveolar spaces expanded.

In IPF models induced by toxins, such as bleomycin, irradiation, asbestos fibers, and CCl_4_, CCl_4_-induced toxicity has been proposed to result in pulmonary injury. Although the primary target organ of CCl_4_ toxicity is the liver, IP injection of CCl_4_ leads to the development in alveolar damage [28, 29]. As olive oil is not harmful, IP and CCl_4_ were dissolved in olive oil for IP application [30, 31]. After inducing the model of pulmonary fibrosis with CCl_4_, it was deposited in many tissues, including the lungs. As noted, CCl_4_ is metabolized by cytochrome-P450 enzyme into two metabolites, CCl_3_^●^ and CCl_3_O_2_^●^ [32]. The rapid reaction of the mentioned metabolites with O_2_ leads to the release of reactive-free radicals. Free radicals, via the oxidation of polyunsaturated fatty acids in cell membranes, help to develop lipid peroxidation. The elevation of MDA levels, as a lipid peroxidation index, leads to intense pulmonary damage [33]. Furthermore, free radicals produced by CCl_4_, by inhibiting antioxidant enzymes such as glutathione peroxidase (GPX), increased oxidized GSH levels and hydrogen peroxide, and formation of reactive oxygen species (ROS). Lower GSH levels protect cell membranes against lipid peroxidation. Thus, following higher oxidative stress, overuse occurs due to reduced GSH levels. In another study, a lower CCl_4_ toxicity by GSH was reported [21]. hMSC-CM prevents the impairment of cell membranes and lipid by reducing the levels of GSH and oxidized glutathione, attenuating myeloperoxidase, and increasing glutathione levels [34–36]. In the present study, while the MDA level in group Fb was more remarkably elevated than that in the control groups (groups C, C.O, and C.Ms), the GSH level was significantly lower than that in the control groups. The GSH and MDA levels were lower in the Fb.Ms group. Therefore, low glutathione and high MDA levels of group Fb demonstrated that CCl_4_ leads to ROS formation and lipid peroxidation, causing lung tissue damage. The elevated GSH level in Fb.Ms compared to that in Fb group and the similar MDA level in the control groups have shown that MSC can prevent lung tissue damage by diminishing ROS formation and lipid peroxidation.

CCl_4_ administration leads to the release of lytic enzymes and cytokines, such as TNF-*α*, following stimulation of the alveolar macrophages. These factors derived from alveolar macrophages exacerbate damage to the bronchial membrane and epithelial cells and impair pulmonary function [37–39]. High TNF-*α* levels, as one of the primary regulatory cytokines of the immune system, can elevate other proinflammatory cytokines and produce oxidant substances by encouraging the migration of neutrophils to damaged parts and releasing proteolytic enzymes derived from them [38, 40]. Many studies have reported that MSC reducer TNF-*α* level by decreasing ROS formation and lipid peroxidation [34, 35, 41–43]. MSC enhances anti-inflammatory responses by enhancing soluble interleukin (IL-1*β*) receptors and IL-10, followed by a decrease in the levels of interferon (IFN-*γ*), TNF-*α,* and IL-2 [14].

In contrast, alveolar macrophages, as the main source of TGF-*β*1, play a multifunctional role in the IPF process by inducing LOX expression in fibroblasts, ECM synthesis, fibroblast proliferation, myofibroblast recruitment, and inflammation promotion [44, 45]. Thus, the high expression of LOX in the IPF process may increase collagen cross-linking [2, 44, 45]. Moreover, active fibroblasts in IPF are created through mechanisms such as epithelial-to-mesenchymal transition (EMT) [46], and TGF-*β*1 is a key factor in the EMT during IPF progression [47]. Hashimoto et al. reported that endothelial cells stimulate the production of numerous fibroblasts in a bleomycin-induced IPF model, and the fundamental mechanism of EMT in endothelial cells is involved in Ras and TGF-*β*1 activation [48]. Additionally, after the activation of TGF-*β*1, habitant fibroblasts can differentiate into myofibroblasts and aggregate in the injured lung tissues [49].

MSC exert anti-inflammatory and antifibrotic effects by secreting cytokine modulators [50]. In one study of bleomycin-induced fibrosis in mice, MSC improved survival by diminishing TNF-*α*, TGF-*β*1, and LOX levels, and finally the inflammation process in IPF [51]. In another study, MSC-derived growth factors were reported to play critical roles in the maintenance of lung permeability and restoration of alveolar epithelial cells following injury [52]. For example, Ionescu et al. revealed that insulin-like growth factor 1 (IGF-1), a growth factor derived from MSC, attenuates lung inflammation [53]. Furthermore, according to Moodley et al., treatment with MSC led to a reduction in lung collagen deposition in the fibrosis of animal models [41]. It is possible that MSC reduces TGF-*β*1 and LOX levels, which in turn reduces collagen deposition and prevents the development of IPF [54]. Interestingly, some studies have reported that MSCs can secrete TGF-*β*1 [51, 55–57]. This phenomenon may explain why they exacerbate bleomycin-induced pulmonary fibrosis when prescribed to mice during the acute fibrotic phase. This finding conflicts with the antifibrotic effects of MSCs [46]. However, Liu et al. revealed the human BM-MSCs isolated from healthy people secreted an extremely high level of TGF-*β*1 compared with MSCs derived from the umbilical cord, which secreted lower levels of TGF-*β*1 [58]. In line with our findings, many studies have showed that the UC-MSC therapy leads to reduced gene expression and protein level of TGF-*β*1 in lung tissue following induced fibrosis [36, 41, 42, 57, 59–61].

## 5. Conclusion

A toxic dose of CCl_4_ may cause severe pulmonary fibrosis by increasing cytokine production, alveolar macrophage infiltration, and ROS formation. hMSC-CM, a potent reducer of TGF-*β*1, LOX, and TNF-*α*, may have a prophylactic effect against CCl_4_-induced toxicity by reducing fibroblast differentiation, collagen deposition, ROS formation, and cytokine production.

## Figures and Tables

**Table 1:** The primer sequences used in the RT-PCR in this study.

Gene	Forward primer	Reverse primer
ACTB	5′-CCCGCGAGTACAACCTTCT-3′	5′-CGTCATCCATGGCGAACT-3′
LOX (rat)	5′-CTCTTCCCAGATCCACAACAA-3′	5′-CCACTCTCCTCTGTGTGCT-3
TGF-*β*1 (rat)	5′-TGCGCCTGCAGAGATTCAAG-3′	5′-AGGTAACGCCAGGAATTGTTGCTA-3′
TNF-*α* (rat)	5′-TGTTCATCCGTTCTCTACCCA-3′	5′-CACTACTTCAGCGTCTCGT-3′

Actin beta: *β*-actin, lysyl oxidase: LOX, transforming growth factor-*β*1: TGF-*β*1 and tumor necrosis factor-*α*: TNF-*α*.

## Data Availability

All data used and analyzed during the current study are included in this manuscript and are available from the corresponding author upon reasonable request.
